# Thermal Performance of T-Shaped Ultra-Thin Vapor Chamber with Double-Sided Heating for LED Automotive Headlamp Cooling

**DOI:** 10.3390/mi16050571

**Published:** 2025-05-12

**Authors:** Yaokang Zhang, Tengqing Liu, Yu Bai, Shuangfeng Wang, Qianxi Zhang, Huifeng Kang

**Affiliations:** 1 College of Ocean Engineering and Energy, Guangdong Ocean University, Zhanjiang 524088, China; zhangyk1129@163.com (Y.Z.); zhangqx@gdou.edu.cn (Q.Z.); 2 School of Chemical and Environmental Engineering, Hunan Institute of Technology, Hengyang 421200, China; baiyuallelo@163.com; 3 Key Laboratory of Enhanced Heat Transfer and Energy Conservation of the Ministry of Education, School of Chemistry and Chemical Engineering, South China University of Technology, Guangzhou 510640, China; 4 School of Aeronautics and Astronautics, North China Institute of Aerospace Engineering, Langfang 065000, China; huifengabc596@163.com

**Keywords:** ultra-thin vapor chamber, double-sided heating, anti-gravity, LED automotive headlamp cooling

## Abstract

High heat flux brings about severe thermal problems for light-emitting diode (LED) automotive headlamps in narrow heat removal spaces, which will degrade their performance and lifespan. This study proposes an easily fabricated and feasible 1.3 mm thick 2D T-shaped in-plane ultra-thin vapor chamber (UTVC) for cooling the high heat flux of LED automotive headlamps. The effects of heating modes, unequal input heat load, and orientations on the thermal performance of the T-shaped UTVC are investigated. The results show that double-sided heating can improve the temperature uniformity of the T-shaped UTVC and reduce the thermal resistance compared to the single-sided heating. The lowest thermal resistances under single-sided and double-sided heating are 1.127 K/W at 12 W and 0.898 K/W at 16 W, respectively. When the total power is identical, the proposed 2D T-shaped UTVC can work effectively at unequal input power. The orientations have a significant impact on the thermal performance of the 2D T-shaped UTVC, and the thermal performance under different orientations changes with anti-gravity state < horizontal state < gravity-assisted state. The proposed T-shaped UTVC can work effectively under diverse operating ranges.

## 1. Introduction

In recent years, light-emitting diodes (LEDs) have been popularly applied to automotive headlamps because of their high light efficiency, long illumination distance, and long lifetime [1,2]. However, only 20% of the input power consumed by LEDs is converted to optical power, while the rest is transformed into waste heat [3]. The heat flux of LED automotive headlamps is high, but the working space is narrow. A large amount of heat will bring about an abrupt chip junction temperature rise, which decreases the electro-optical conversion efficiency, drifts the wavelength of the light, and shortens the lifespan of the LED [4]. Therefore, effectively removing heat in narrow spaces has become an important issue in the development of LED automotive headlamps.

Various thermal management technologies have been developed to address the heat dissipation needs for LED cooling. Among them, heat pipes and vapor chambers have emerged as one of the most promising technologies for cooling LED automotive headlamps due to their high thermal conductivity, flexible shape design, and compactness. For typical LED cooling, Tang et al. [5] fabricated a ∅16 mm and L 80 mm columnar heat pipe for cooling 42 high-power LED devices. Sintered copper powder was used as a wick structure. At 2800 mA, the average junction temperature of the columnar heat pipe was 101.6 °C, which was lower than the copper heat sink by 15.4 °C. Pekur et al. [6] proposed a heat sink with eight radial heat pipes and 20 heat exchanger rings. The sizes of heat pipes were ∅6·mm and L 250 mm. The simulation results showed that at 500 W, the junction temperature was 85.5 °C and the thermal resistance was 0.131 K/W. Xiao et al. [7] proposed an automatic heat pipe heat sink for 12 W high-power LED cooling. The sizes of heat pipes were ∅6·mm and L 128 mm. The simulation results showed that at 12 W, the junction temperature was 79.15 °C and the thermal resistance was 5.953 K/W. Lin et al. [8] developed a 180 × 120 × 4 mm^3^ aluminum plate oscillating heat pipe (OHP) for LED cooling. They found that at 64 W, the temperature of the LED with the plate OHP was maintained at 61.40 °C, and the plate OHP LED heat sinks showed better performance than aluminum plates with the same size regardless of orientation variation. Chang et al. [9] developed 90 × 60 × 5 mm^3^ 3D printed aluminum flat-plate oscillating heat pipes for LED cooling, and AlSi_10_Mg powders were melted and solidified to form the chamber of the flat OHP. They found that when the LEDs were in contact with the 3D printed flat OHP and the power was 40 W, the temperature of the LEDs was maintained at 60 °C and their lifespan was prolonged. Xu et al. [10] proposed rectangular thermosyphons with non-uniform height for high-power LED cooling. The sizes of the thermosyphons were 55 × 55 × 5 mm^3^ with fin heights of 45 mm. The simulation results showed that the maximum heat transfer rates of type-a, type-b, and type-c thermosyphons were 19 W, 17 W, and 22 W concerning the temperature threshold of 85 °C, respectively. Li et al. [11] developed a loop heat pipe (LHP) with dual condensers for high-power LED cooling. The evaporator sizes were 30 × 30 × 15 mm^3^, the condenser sizes were 120 × 80 × 50 mm^3^, and the inner diameters of the vapor and liquid lines were 5 mm. They found that the junction temperature of a 50 W LED chip was kept below 85 °C. Ušakovs et al. [12] created an advanced loop heat pipe (LHP) for 80 W LED cooling, and butane was used as a working fluid. The 350 mm long and 15 mm diameter integrated LHP showed high resistance in the anti-gravity orientation, which was higher than in a gravity-assisted orientation (0.8 K/W vs. 0.03 K/W). Wang et al. [13] proposed a 90 × 90 × 3.0 mm^3^ vapor chamber for 30 W high-power LED cooling. It is found that at 30 W, the mean temperature of the LED vapor chamber was 77 °C at 60 s, while the mean temperature of the LED aluminum plate was over 80 °C at 40 s. Tang et al. [14] developed a 150 × 150 × 2.8 mm^3^ aluminum-based integrated vapor chamber heat sink, and parallel and orthogonal microgrooves were used as a wick structure. The finding was that at 3200 mA (approximately 130 W), the junction temperature of the vapor chamber heat sink was 91.71 °C, which is 13.04 °C lower than that of the copper heat sink.

As for LED automotive headlamp cooling, many cooling methods have also been proposed, such as air cooling, liquid cooling, and heat pipe/vapor chamber cooling. Several studies have focused on heat pipes and vapor chamber-based heat sinks because of their high thermal conductivity and compact and flexible design. Wang et al. [15] developed a conventional heat pipe heat sink for LED headlamp cooling, and the length of the heat pipe was 120 mm and the diameter was 6 mm. At 25 W, when the filling ratio was 10%, the evaporation section length was 30 mm, the adiabatic section length was 40 mm, and the condensation section length was 50 mm, the heat pipe heat sink had the best cooling performance. Zhao et al. [16] also fabricated a conventional heat pipe heat sink for LED headlamp cooling, and the length of the heat pipe was 120 mm and the diameter was 8 mm. The Al_2_O_3_/water nanofluids were used as the working fluid. When the heat loads ranged from 5 to 25 W, the junction temperature could be controlled below 113 °C. Nowadays, the LED automotive headlamps’ heat removal space has become narrower; thus, small-area heat pipes and vapor chambers have been developed. Huang et al. [17] developed a grooved heat pipe for LED headlamp cooling, and the size of the grooved heat pipes was 63.5 × 8 × 4 mm^3^. At 30 W, the measured junction temperature was 103.67 °C. Tang et al. [18] developed two flattened heat pipes with a size of 55 × 3 × 2 mm^3^ for LED headlamp cooling. Two LED headlamps were installed on both sides of the ceramic-based PCB (CBPCB) substrate, and the two flattened heat pipes were welded to the left and right sides of the CBPCB. At 34 W, the junction temperature was 100.32 °C. Jian et al. [19] developed a 3D T-shaped vapor chamber by welding a circular vapor chamber and flattened heat pipe. The sizes of the circular vapor chamber were ∅39.5 mm and H 4 mm, and the size of the flattened heat pipe was 57.3 × 12.5 × 2.5 mm^3^. Two heat sources with a size of 12.5 × 15 mm^2^ were applied on both sides of the evaporation section on the flattened heat pipe, and the circular vapor chamber served as the condensation section. The minimum thermal resistance of the 3D T-shaped vapor chamber was 0.401 K/W at 15.84 W. Lu et al. [20] also fabricated a 3D T-shaped vapor chamber by combining a circular vapor chamber and a flattened heat pipe. The sizes of the circular vapor chamber were ∅40 mm and H 4 mm, and the length of the flattened heat pipe was 57 mm with a diameter of 8 mm. The flattened thickness was 3.5 mm and 2.3 mm, wherein the length of the flattened thickness of 3.5 mm was 17 mm. Two heat sources with a size of 20 × 8 mm^2^ had contact on both sides of the evaporation section on the flattened heat pipe, and the circular vapor chamber served as the condensation section. The lowest thermal resistance of the 3D vapor chamber was 0.125 K/W at 50 W.

The aforementioned 3D vapor chamber for LED automotive headlamp cooling shows good thermal performance. However, the manufacturing process of the 3D T-shaped vapor chamber is complicated. Moreover, a thinner vapor chamber is needed for a narrower heat removal space. In this study, to simplify the manufacturing process and develop a thinner vapor chamber for LED headlamp cooling, a novel 2D T-shaped in-plane ultra-thin vapor chamber (TUTVC) with a thickness of 1.3 mm has been developed. The effect of heating modes, unequal input power, and orientations on the thermal performance of the novel 2D T-shaped UTVC has been systematically investigated. This study will provide some useful guidelines for designing UTVC applications in LED automotive headlamp cooling.

## 2. Experiments

### 2.1. Description of the T-Shaped In-Plane UTVC

**Figure 1:**
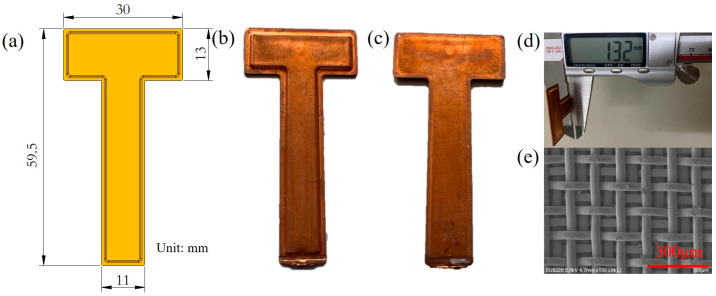
Two-dimensional T-shaped in-plane UTVC. (**a**) Schematic diagram of the 2D T-shaped UTVC; (**b**) photography of the bottom side of the T-shaped UTVC; (**c**) photography of the upper side of the T-shaped UTVC; (**d**) thickness of the T-shaped UTVC; (**e**) wick structure inside the T-shaped UTVC.

**Figure 2:**
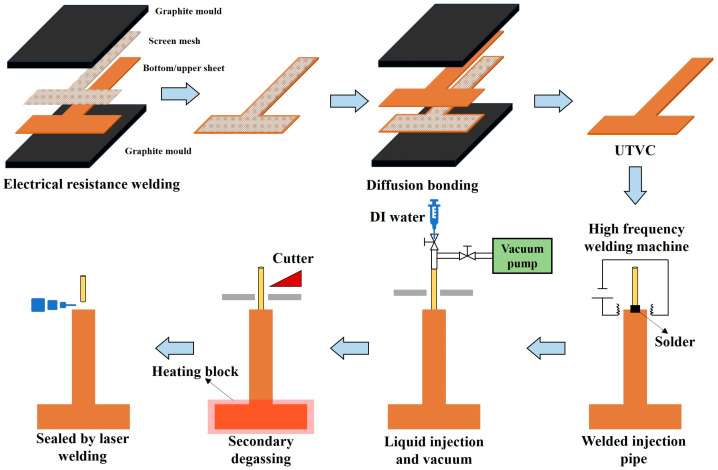
The manufacturing process of the 2D T-shaped UTVC.

In the study, a 1.3 mm thick 2D T-shaped in-plane UTVC was developed. The schematic diagram and photo of the 2D T-shaped in-plane UTVC and the wick structure inside the T-shaped UTVC are shown in Figure 1. The 2D T-shaped in-plane UTVC contained a bottom sheet, an upper sheet, a wick structure, and a charging tube. They were all made of red copper (C1100). The wall thicknesses of the bottom sheet and upper sheet were both 0.3 mm. Figure 2 shows the manufacturing process of the 2D T-shaped in-plane UTVC. As shown in Figure 2, the bottom sheet was stamped by stainless-steel molds to fabricate the chamber. The internal height of the chamber was 0.7 mm. Two layers of 180 in^−1^ screen meshes were bonded on the inner wall of both the bottom and upper sheets by electrical resistance welding as the wick structure. The SEM photo of the two layers of 180 in^−1^ screen mesh wick is shown in Figure 1e, and the structure parameters are listed in Table 1. The wire diameter of the 180 in^−1^ mesh was 0.05 mm, the wire spacing was 0.0911 mm, and the thickness of the two layers of the 180 in^−1^ mesh was 0.2 mm. After mesh wick welding on the bottom and upper sheets, the two sheets were aligned and clamped by graphite molds. Then, the two sheets were welded together by diffusion bonding under 750 °C for 1.5 h in a 95% N_2_ and 5% H_2_ atmosphere. The charging tube was welded to the vapor chamber by high-frequency welding.

Deionized water was used as a working fluid, and the filling ratio was 100% of the pore volume inside the mesh wick. The non-condensable gas in the vapor chamber was removed via the coarse and fine evacuation and heat-degassing process, and the vacuum degree was less than 10 Pa. Ultimately, the whole charging tube was cut by the cutter and sealed through laser welding.

As shown in Figure 1a, the size of the horizontal stripe of the T-shaped UTVC was 30 × 13 mm^2^, and the size of the vertical stripe was 46.5 × 13 mm^2^. The thickness of the T-shaped UTVC was 1.32 mm, as shown in Figure 1d.

### 2.2. Experimental Setup and Test Conditions

**Figure 3:**
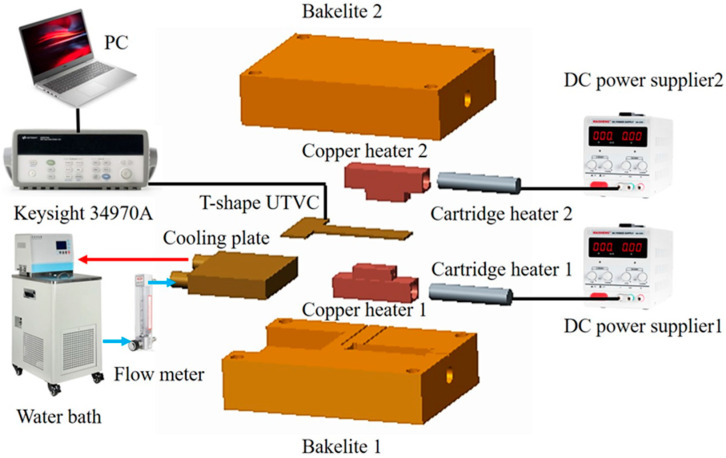
The schematic diagram of the experimental setup.

The schematic diagram of the experimental setup is shown in Figure 3. As shown in Figure 3, the experimental setup consisted of a heating part, a cooling part, a data acquisition system, and an insulation part.

The heating part consisted of two direct current power suppliers (Maisheng MS-605DS with a voltage and current meter and a measurement uncertainty of ±0.01 V and ±0.001 A, Maihao Electronic Co., Ltd., Dongguan, China), two copper heaters with a heating area of 20 × 8 mm^2^, and two cartridge heaters. Cartridge heater 1 was inserted into copper heater 1, and cartridge heater 2 was inserted into copper heater 2. Copper heater 1 came into contact with the evaporation section on the bottom sheet, and copper heater 2 came into contact with the evaporation section on the upper sheet. The two copper heaters simulated the heat of the LED automotive headlamps. When the input power of copper heater 1 and copper heater 2 was x W and y W, respectively, the total input power Q_tot_ applied on the 2D T-shaped UTVC was xW + yW. There were two heating modes in our study, namely single-sided heating and double-sided heating. The single-sided heating was when x or y were to equal 0, and double-sided heating was when x and y > 0.

The cooling part consisted of a thermostatic water bath (DC1010 with a temperature control uncertainty of ±0.05 °C, Yisheng Experiment Instrument Co., Ltd., Guangzhou, China), a rotor flow meter (LZB-6WB with a measurement uncertainty of ±2% at full scale, Darhor Instrument Inc., Hangzhou, China), connection pipes, and a cooling plate with a cooling area of 40 × 40 mm^2^. The cooling plate was in contact with the condensation section of the bottom sheet. The constant-temperature cooling water was pumped to the cooling plate, and the flow rate was regulated by the control valve within the rotor flow meter.

**Figure 4:**
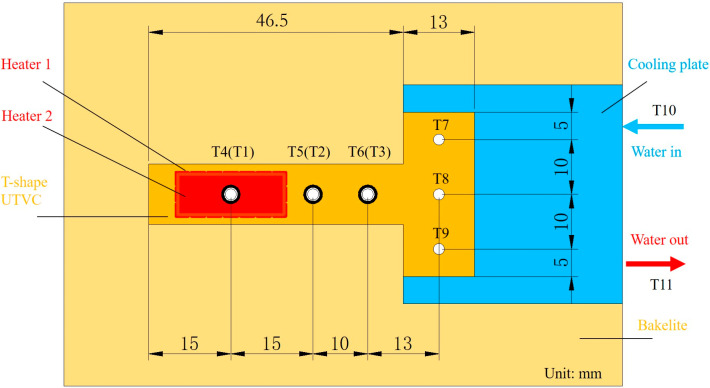
Thermocouple distribution on the 2D T-shaped UTVC.

The data acquisition system measured and recorded the temperatures of the UTVC test system. It consisted of a personal computer, Omega K-type thermocouples with a measurement uncertainty of ±0.1 °C, OMEGA Measurement Technology Co., Ltd., Shanghai, China, and a data acquisition apparatus (a Keysight 34970A and a module 34901A, Keysight Technologies, Beijing, China). The thermocouple distribution on the T-shaped UTVC test system is shown in Figure 4. As shown in Figure 4, T1–T3 contact with the bottom sheet, T4–T9 contact with the upper sheet, and T10 and T11 were used to monitor the inlet and outlet water temperatures. T1 and T4 were in the center of copper heaters 1 and 2, respectively. T2, T3, T5, and T6 were on the adiabatic section of the UTVC. T7–T9 were on the condensation section of the UTVC. The insulation part included Bakelite 1 and Bakelite 2. They insulated the whole UTVC and two copper heaters from the room. Hence, heat loss was reduced. Furthermore, a thin layer of thermal grease with a thermal conductivity of 4.0 W/(m·K) was smeared on the copper contact surface to reduce the thermal resistance.

It can also be seen from Figure 3 and Figure 4 that the evaporation section was at the end of the vertical stripe of the T-shaped UTVC, and the whole horizontal stripe of the T-shaped UTVC served as the condensation section, which expanded the condensation area. The liquid in the wick of the evaporation section absorbed waste heat from LED automotive headlamps and vaporized and flowed in the vapor channel to the condensation section. The heat transferred to the heat sink, and the vapor condensed. The condensate flowed back to the evaporation section by the capillary force of the two layers of screen mesh wick. The 2D T-shaped vapor chamber was easy to manufacture and compact.

**Figure 5:**
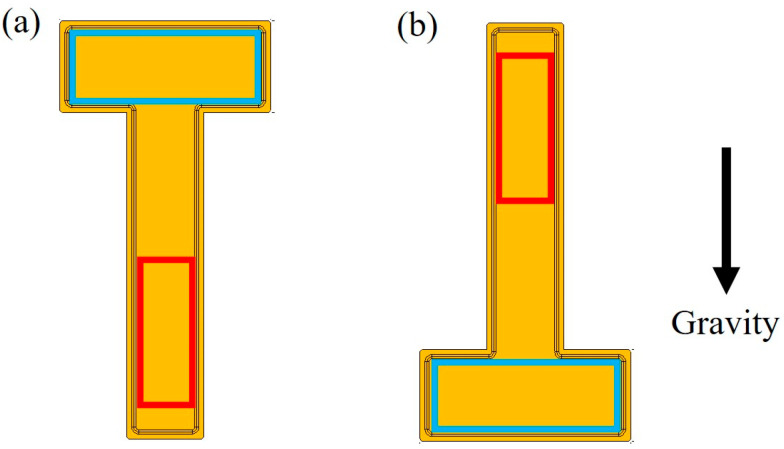
Schematic diagram of the orientation test of the 2D T-shaped UTVC: (**a**) gravity-assisted state; (**b**) anti-gravity state.

In the test, the room temperature remained within 25 ± 1 °C by an air conditioner. The cooling water temperature was set to 35 °C, and the water flow rate was 0.8 L/min. The effect of heating modes on the thermal performance of the 2D T-shaped UTVC was conducted, and the UTVC test system was placed horizontally, as shown in Figure 3. Also, to assess the effect of orientation on the thermal performance of the 2D T-shaped UTVC, the single-sided heating mode under a gravity-assisted state and anti-gravity state, as shown in Figure 5, was used.

### 2.3. Data Reduction and Uncertainty Analysis

Thermal resistance is a key technical indicator to evaluate the thermal performance of heat pipes and vapor chambers under thermal equilibrium. In this study, the thermal resistance of the 2D T-shaped vapor chamber is calculated by Equation (1):

(1) $$R_{VC}=\frac{T_{eva}-T_{cond}}{Q}=\frac{(T_{1}+T_{4})/2-\left(T_{7}+T_{8}+T_{9}\right)/3}{Q}$$

where *Q* is the input power of the DC power supply and *T_eva_* and *T_cond_* are the evaporator and condenser temperatures, respectively, averaged over time. Both evaporator and condenser temperatures are usually referred to as temperatures measured on the vapor chamber outer surface [21]. T1, T4, T7, T8, and T9 were the temperatures located on the 2D T-shaped UTVC, as shown in Figure 4.

The uncertainty analysis of the thermal resistance can be expressed as follows [22]:

(2) $$\frac{\delta y}{y}=\frac{\sqrt{\sum_{i=1}^{n}{\left(\frac{\partial y}{\partial x_{i}}\delta x_{i}\right)}^{2}}}{y}$$

where *x_i_* and *y* are the independent variable and function of *x_i_*, respectively. The uncertainty of the thermal resistance in this study was less than 7%.

## 3. Results and Discussion

### 3.1. Effect of Heating Modes

**Figure 6:**
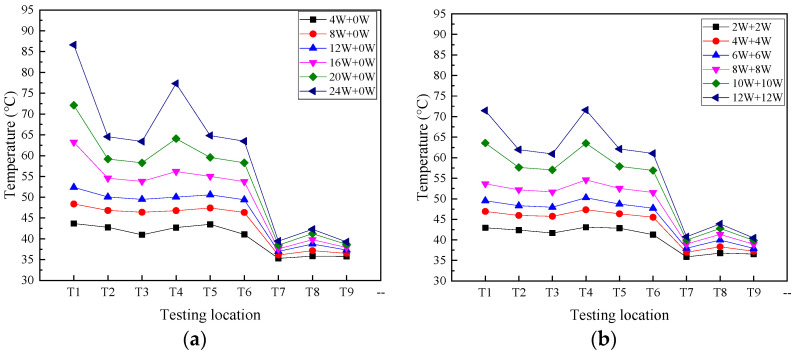
Temperature distribution on 2D T-shaped UTVC under different heating modes: (**a**) single-sided heating; (**b**) double-sided heating with equal input power.

Figure 6 depicts the temperature distribution on the 2D T-shaped UTVC under two heating modes. In this section, the single-sided heating mode is *Q* = *xW* + *yW*, where y = 0, and the double-sided heating mode is *Q* = *xW* + *yW*, where *x* = *y*. The UTVC test system is placed in a horizontal state, and the total input power starts from 4 W to 24 W with a step of 4 W. It can be seen from Figure 6 that by increasing the input heat load, the temperatures on the T-shaped UTVC increase under the two heating modes. Moreover, the temperature uniformity under double-sided heating is better than that under single-sided heating. At the same input heat load, the temperature difference between the heat source temperatures (T1 and T4) under single-sided heating is higher than that under double-sided heating. At 4 W, 8 W, 12 W, 16 W, 20 W, and 24 W, the temperature differences between T1 and T4 are 0.98 °C, 1.59 °C, 2.37 °C, 7.02 °C, 8.03 °C, and 9.30 °C, respectively, under single-sided heating, while the temperature difference between T1 and T4 is within 1 °C under double-sided heating. It can also be seen that at the same input heat load, the adiabatic section temperatures T2, T3, T5, and T6 under single-sided heating are higher than those under double-sided heating, but the condensation temperatures T3, T5, and T6 under single-sided heating are less than those under double-sided heating.

This can be attributed to the heating area difference between the two heating modes. The heating area in single-sided heating mode is half of that in double-sided heating mode. At single-sided heating, the liquid on the heating side evaporates, and the other side does not evaporate, while at double-sided heating, the liquid on the two heating surfaces evaporates simultaneously. At the same input power, the enlarged surface under double-sided heating can increase the evaporation rate and shorten the effective liquid backflow path (i.e., flow cross-sectional area) to the evaporation section, which could significantly promote the circulation efficiency and improve the temperature uniformity. The effective liquid backflow path of single-sided heating is larger than that of double-sided heating; therefore, the flow resistance is large, which makes the condensate accumulate at the condensation section and lower the condensation temperature.

**Figure 7:**
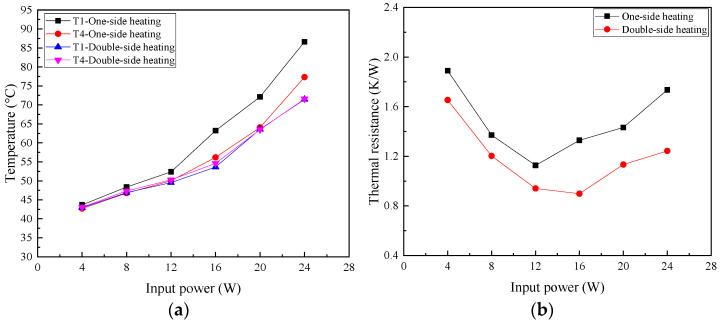
T1 and T4 on 2D T-shaped UTVC and thermal resistance of 2D T-shaped UTVC under different heating modes: (**a**) T1 and T4 vs. input power; (**b**) thermal resistance vs. input power.

Figure 7 presents T1 and T4 on 2D T-shaped UTVC and the thermal resistance of T-shaped UTVC under two heating modes. It can be seen from Figure 7a that the temperature difference in T1 and T4 under single-sided heating is much larger than that under double-sided heating. In addition, the temperature difference increases under single-sided heating after 12 W since partial dry-out occurs. The temperature difference under double-sided heating is small, within 1 °C, due to a larger evaporation area and a much more effective liquid flow cross-section area in the wick.

Figure 7b shows that the thermal resistance under double-sided heating is smaller than that under single-sided heating. This is because the enlarged evaporation area under double-sided heating increases the temperature uniformity, i.e., decreases the heat source temperature and increases the condensation temperature. Moreover, the thermal resistances under two heating modes decrease with the increasing input power, reach the minimal point, and then increase. At low input power, as the input power increases, the evaporative liquid film becomes thinner due to the increasing evaporation rate, which will decrease the thermal resistance. When the thermal resistance reaches the smallest point, partial dry-out will occur with a further increase in the input power, which will increase the thermal resistance. The minimum thermal resistances under single-sided and double-sided heating are 1.127 K/W at 12 W and 0.898 K/W at 16 W, respectively. The partial dry-out limit under double-sided heating is larger than that under single-sided heating.

### 3.2. Effect of Unequal Input Power

**Figure 8:**
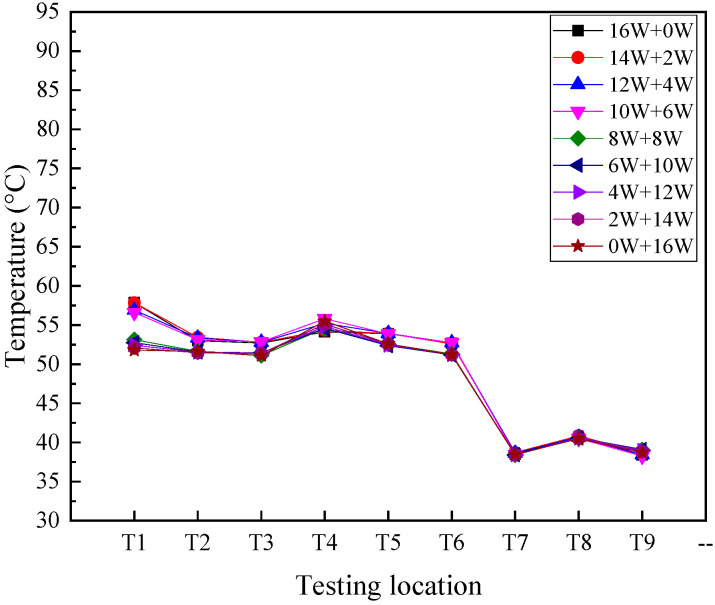
Temperature distribution on 2D T-shaped UTVC under double-sided heating with unequal input heat load.

The effect of unequal input power on the temperature distribution of 2D T-shaped UTVC under double-sided heating is shown in Figure 8. The total input power is 16 W. The input powers of copper heaters 1 and 2 are (i) 16 W and 0 W, (ii) 14 W and 2 W, (iii) 12 W and 4 W, (iv) 10 W and 6 W, (v) 8 W and 8 W, (vi) 6 W and 10 W, (vii) 4 W and 12 W, (viii) 2 W and 14 W, and (ix) 0 W and 16 W, respectively. As shown in Figure 8, at different input power distributions, the condensation temperatures (T7–T9) remain the same. When the input power of copper heater 1 is larger than that of copper heater 2, the heat source temperatures and adiabatic temperatures are almost the same. When the input power of copper heater 1 is smaller than or equal to that of copper heater 2, the heat source temperatures and adiabatic temperatures are also almost the same. But the temperature uniformity is better when the input power of copper heater 1 is smaller than or equal to that of copper heater 2. The main reason is that the input power is medium, and the capillary force is sufficient to return the condensate. In addition, the flow resistance to heat source region 1 is larger than that to heat source region 2, which makes the condensate flowing back easier to heat source region 2.

It is indicated that the 2D T-shaped UTVC proposed in the study can work effectively at unequal power distribution for the multi-LED heat dissipation application when the total power is identical.

### 3.3. Effects of Test Orientation

Gravity has been considered to affect thermal performance when an automobile goes uphill and downhill. Moreover, inertial forces during acceleration or braking are much greater than the gravitational forces caused by driving up or down a normal slope.

The capillary force of the wick must be greater than the frictional force of vapor and liquid and the gravitational force [23]. When the UTVC is placed in a horizontal state, the effect of gravitational force is ignored. At the anti-gravity state, the gravitational force will cause a pressure drop in the condensate flow. At the gravity-assisted state, the gravitational force will accelerate the condensate returning to the evaporation section.

**Figure 9:**
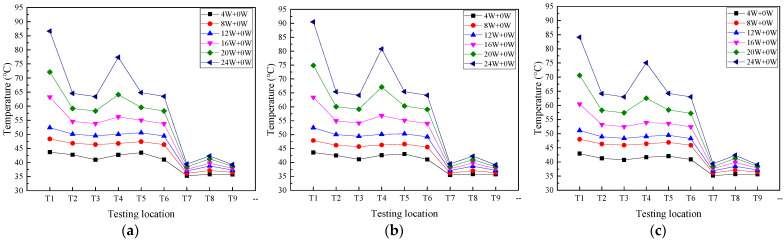
Temperature distribution on 2D T-shaped UTVC with single-sided heating under different orientations: (**a**) horizontal state; (**b**) anti-gravity state; (**c**) gravity-assisted state.

In this study, the effect of orientations on thermal performance has been conducted, and the temperature distribution on 2D T-shaped UTVC with single-sided heating under the horizontal state, anti-gravity state, and gravity-assisted state is shown in Figure 9.

As seen in Figure 9, at different orientations, the condensation temperatures (T7–T9) remain the same. When the input power is less than 16 W, the temperature distribution under the anti-gravity state is almost the same as that under the horizontal state, but when the input power exceeds 16 W, the temperatures on the heat source and adiabatic section are larger than those under the horizontal state. It is indicated that at low and medium input heat load, the capillary force can counteract the gravity force and the 2D T-shaped UTVC maintains good performance, but at high input power, the capillary force cannot counteract the gravity force and the condensate cannot return to the evaporation section rapidly, resulting in the temperature rising on the heat source and adiabatic section.

It can also be seen from Figure 9 that with the assistance of gravity, at all input powers, the temperature distribution on the heat source and adiabatic section under the gravity-assisted state is lower than that under the horizontal state, which greatly improves the thermal performance of the 2D T-shaped UTVC.

**Figure 10:**
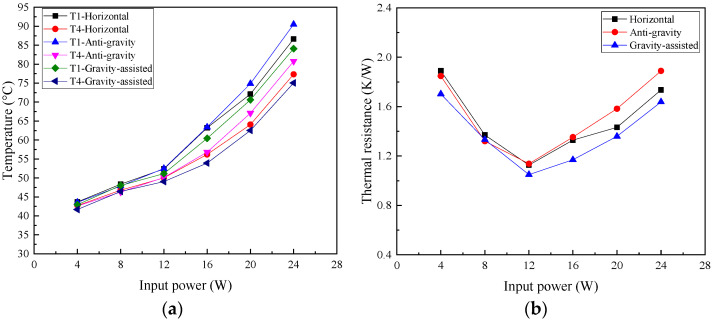
T1 and T4 on 2D T-shaped UTVC and thermal resistance of 2D T-shaped UTVC under different orientations: (**a**) T1 and T4 vs. input power; (**b**) thermal resistance vs. input power.

The T1 and T4 on 2D T-shaped UTVC and thermal resistance of T-shaped UTVC under different orientations are presented in Figure 10. As shown in Figure 10a, the highest temperature T1 at 24 W under the horizontal state, anti-gravity state, and gravity-assisted state is 86.63 °C, 90.49 °C, and 84.09 °C, respectively. At high input power, the gravity-assisted state effectively decreases the highest temperature, but the anti-gravity state increases the highest temperature.

As seen from Figure 10b, when the input power is lower than 16 W, the curve of thermal resistance under the anti-gravity state coincides with that under the horizontal state. When the input power exceeds 16 W, the thermal resistance under the anti-gravity state is larger than that under the horizontal state due to the insufficient capillary force at high input power. With the assistance of gravity, the thermal resistance under the gravity-assisted state is always smaller than that under the horizontal state. Overall, the proposed 2D T-shaped UTVC shows good thermal performance under different orientations.

### 3.4. Comparison of Other Studies

The thermal performance comparisons of the 2D T-shaped UTVC in this study and the 3D T-shaped VC in the references are listed in Table 2.

The thermal resistance of the 2D T-shaped UTVC is larger than that in Refs. [19,20] because of its ultra-thin thickness of 1.3 mm, which is much smaller than the thicknesses of 4 mm in Refs. [19,20]. This reason will greatly increase the frictional force and increase thermal resistance. Due to its ultra-thin thickness and suitable thermal performance, the proposed 1.3 mm thick 2D T-shaped UTVC can be applied in a compact space of automotive LED headlamp cooling.

## 4. Conclusions

In this study, a novel 1.3 mm thick 2D T-shaped UTVC has been proposed and developed for high-power LED automotive headlamp cooling in a narrow space. The effects of heating modes, unequal input power, and orientations on the thermal performance of the T-shaped UTVC are investigated. The following main conclusions can be drawn:

(1) The double-sided heating with equal power can improve the temperature uniformity of the T-shaped UTVC and reduce the thermal resistance of the T-shaped UTVC compared to the single-sided heating. The lowest thermal resistances under single-sided and double-sided heating are 1.127 K/W at 12 W and 0.898 K/W at 16 W, respectively.
(2) When the total power is identical, the proposed 2D T-shaped UTVC can work effectively at unequal input power under double-sided heating.
(3) The thermal performance of the 2D T-shaped UTVC under the anti-gravity state is similar to that under the horizontal state at low and medium input power but worse at high input power. The thermal performance of the 2D T-shaped UTVC under the gravity-assisted state is significantly improved by the assistance of gravity.

The proposed easily fabricated and compact 1.3 mm thick 2D T-shaped UTVC shows good thermal performance under diverse operating ranges.

## Figures and Tables

**Table 1:** Parameters of the two layers of the 180 in^−1^ screen mesh wick.

Parameters	Values
Mesh size *M*/mm	7.087
Wire diameter *d_s_*/mm	0.05
Wire spacing *w_s_*/mm	0.0911
Effective capillary radius *r_eff_*/mm	0.0490
Porosity *ε*/%	70.8
Permeability *K*/mm^2^	8.511 × 10^−5^
Mesh thickness *th_w_*/mm	0.20

**Table 2:** The thermal performance comparisons of other studies.

Sizes of T-Shaped VCs	Thermal Performance
This study: 2D T-shaped UTVC, horizontal stripe 30 × 13 × 1.3 mm^3^, vertical stripe 46.5 × 13 × 1.3 mm^3^	Single-sided heating: R_min_ = 1.127 K/W, Q_max_ = 24 W Double-sided heating: R_min_ = 0.898 K/W, Q_max_ = 24 W
Ref. [19]: 3D T-shaped vapor chamber, circular vapor chamber ∅39.5 mm and H 4 mm, flattened heat pipe 57.3 × 12.5 × 2.5 mm^3^	R_min_ = 0.401 K/W, Q_max_ = 15.84 W
Ref. [20]: 3D T-shaped vapor chamber, circular vapor chamber ∅40 mm and H 4 mm, flattened heat pipe ∅8 mm and L 17 mm flattened to 3.5 mm, ∅8 mm, and L 39 mm and flattened to 2.3 mm	R_min_ = 0.125 K/W, Q_max_ = 50 W

## Data Availability

The datasets generated and analyzed during the current study are available from the corresponding author upon reasonable request.
